# Supplementary material for: CAR-Modified Vγ9Vδ2 T Cells Propagated Using a Novel Bisphosphonate Prodrug for Allogeneic Adoptive Immunotherapy
Source: Int J Mol Sci. 2023 Jun 29;24(13):10873. doi: 10.3390/ijms241310873 (PMC10341429; doi:10.3390/ijms241310873)
Supplement: Supplementary file 1 [file ijms-24-10873-s001.zip › ijms-2435597-supplementary.pdf]

SUPPLEMTAL INFORMATION

SUPPLEMENTALY FIGURES

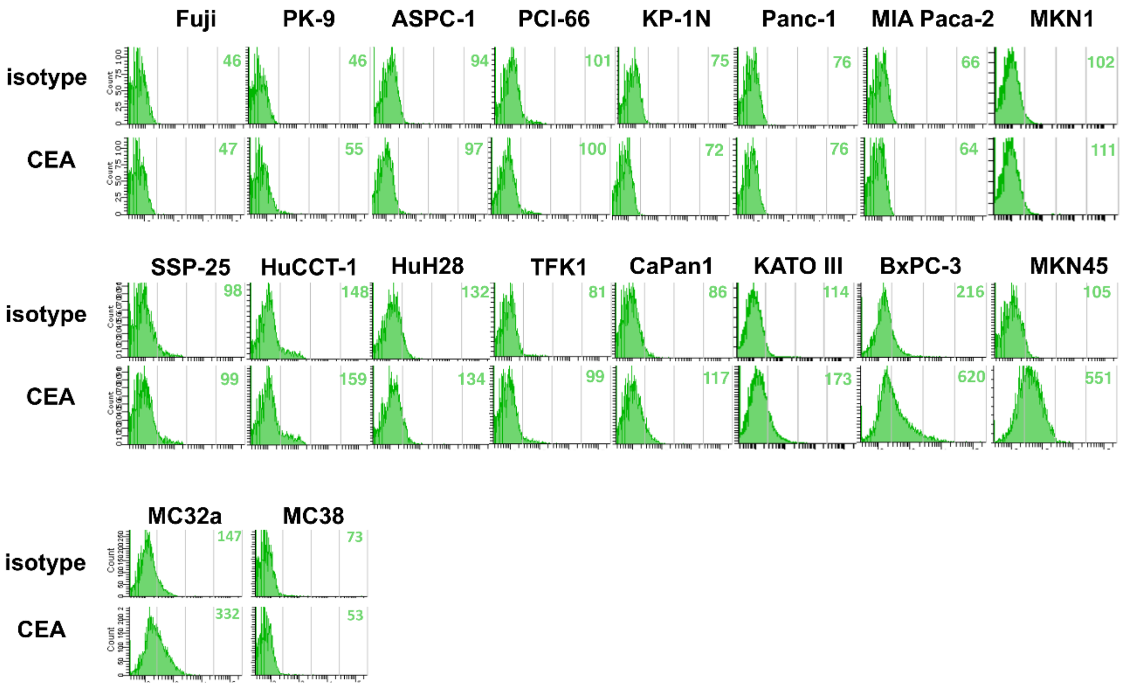


**Figure S1.    Cell surface expression of CEA on various tumors.**

Tumor cell lines were stained with anti-CEA (FITC-anti-CD66e, Clone:REA876) or isotype control mAb (clone:QA16A12), and subjected to FACS analysis.

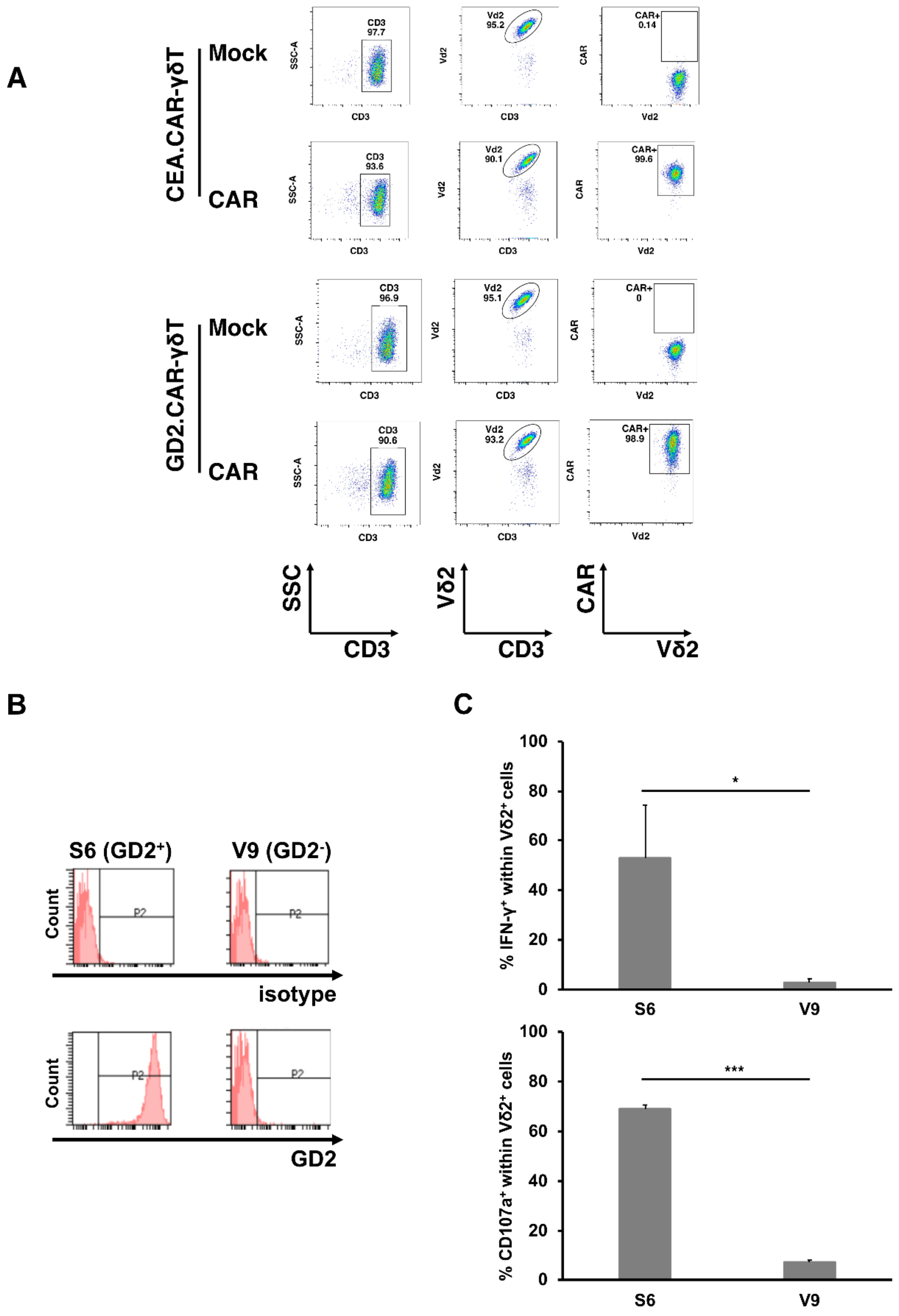


**Figure S2. GD2 specific CAR-γδ T cells.** (A) FACS profiles of various γδ T cell preparations for adoptive transfer. Representative of CEA.CAR or GD2.CAR

expression on Vδ2$^+$ T cells on day 10. γδ T cell transduced with CEA.CAR or GD2.CAR were stained with biotin-CEA (CEA.CAR-γδ T cells) or rabbit-anti-human IgG kappa LC (GD2.CAR-γδ T cells) followed by streptavidin PE-conjugated or goat-anti-rabbit IgG Alexa 488 conjugated, respectively. These cells subsequently stained with V450-anti-human CD3 (Clone: UCHT1) and APC-anti-human TCR Vδ2 (Clone: B6), and subjected to FACS analysis. Mock-γδ T cells stained similarly served as controls. (B) FACS analysis of GD2 expression using PE-anti-human GD2 (Clone:14G2a) on S6 (human melanoma tumor cells) and V9 (human melanoma tumor cells). Isotype matched mAb (Clone MOPC-173) was used as negative control. (C) IFN-γ production and CD107a expression were assayed by using 13 days cultured GD2.CAR- γδ T cells ($3 \times 10^5$) cells co-culture with GD2$^+$ (S6) ($3 \times 10^5$) or GD2$^-$ (V9) ($3 \times 10^5$) human melanoma tumor cell lines. Error bars represent SD of the mean. *$P < 0.05$, ***<0.001.

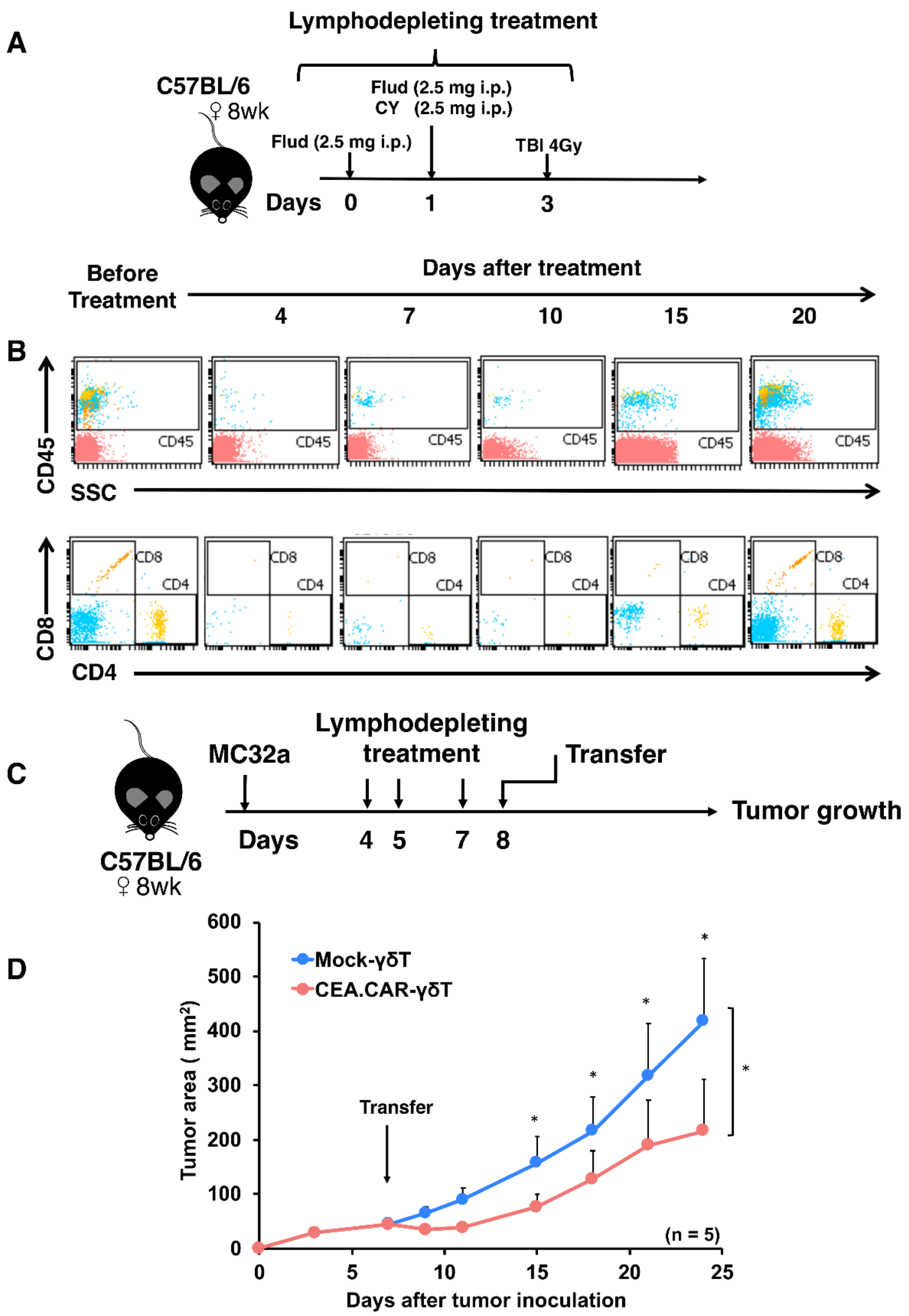


**Figure S3. Anti-tumor function of CEA.CAR-γδ T cells transferred into wild-type host received lymphodepletion.**

(A) A schematic representation of lymphodepleting treatment of mice and cell population dynamics in these mice. C57BL/6 mice (n = 5) were received i.p. injection of fludarabine (Flud) (SANOFI, 2.5 mg/500 μL/mouse), cyclophosphamide (CY) (SHIONOGI, 2.5 mg/500 μL/mouse) and 4-Gy total body irradiation (TBI) under anesthesia at indicated time points. (B) PBMCs (n = 3) were collected from these mice by retro-orbital bleeding, pooled, and subjected to flow cytometry after staining with PE-anti-mouse CD4 (Clone: RM4-5), APC-anti-mouse CD8 (Clone: 53-6.7), and V500-anti-mouse CD45 (Clone: 30-F11). (C) A schematic representation of lymphodepleting treatment followed by adoptive transfer with CEA.CAR-γδ T cells into tumor-bearing mice and tumor growth curves of these mice (n = 5). C57BL/6 mice were injected s.c. with ($2.5 \times 10^6$) MC32a (mouse colon cancer cell line MC38 transduced with CEA) tumors and subsequently received lymphodepleting preconditioning as indicated followed by i.v. transfer with CEA.CAR-γδ T cells and Mock-γδ T cells ($1 \times 10^7$ cells) on day 8. (D) Tumor areas were measured by a caliper using the formula (length × width) at the indicated time points. Error bars represent SD of the mean. *$P < 0.05$. A representative result from 2 independent experiments is shown.

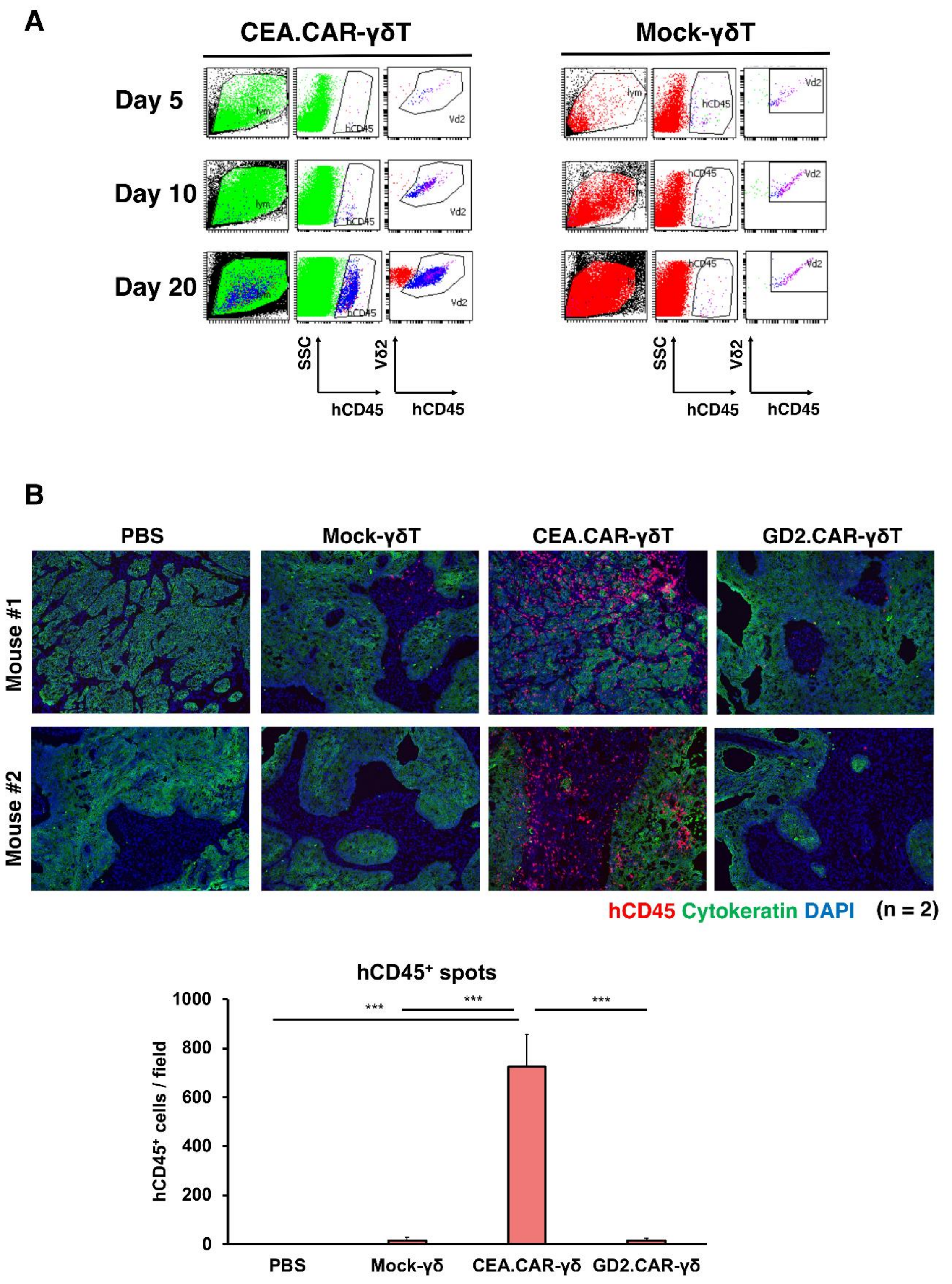


**Figure S4. An antigen-specific accumulation of CEA.CAR-γδ T cells within tumor tissues**

NOG mice were inoculated s.c. with BxPC-3 ($5 \times 10^6$ cells) followed by i.v. injection with CEA.CAR-γδ T cells, GD2.CAR-γδ T cells, Mock-γδ T cells ($5 \times 10^6$ cells) and PBS. (A) Flow cytometry analysis of transferred CEA.CAR-γδ T cells and Mock-γδ T cells in tumor tissues from BxPC-3-bearing NOG mice (n = 2) on day 5, day 10 and day 20. Single cell suspensions of tumor tissues were pooled and stained with V450 anti-human CD45 (Clone: HI30) and FITC-anti-human TCR Vδ2 (Clone: B6). (B) Tumor tissues were collected on day 20 after the transfer as above and subjected to fluorescence IHC (n = 2) using PE-anti-human CD45 (Clone: HI30) (red), Pan-Cytokeratin (Clone: AE1/AE3) (green) and DAPI (blue). $hCD45^+$ cells in the field were counted and plotted in bar graph. Error bars represent SD of the mean. ****P<0.001*

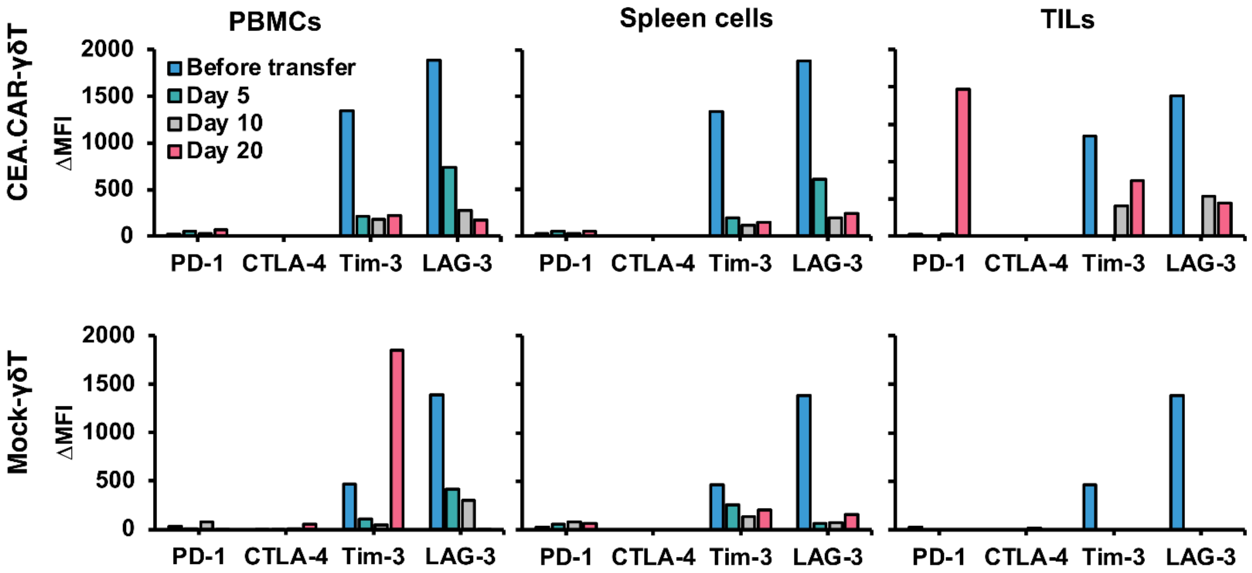


**Figure S5. Expression of co-inhibitory receptors on CEA.CAR-γδT cells in vivo**

NOG mice (n = 4) were inoculated s.c. with BxPC-3 ($4 \times 10^6$ cells) followed by i.v. injection with CEA.CAR-γδ T cells or Mock-γδ T cells ($5 \times 10^6$ cells). Pooled single cell suspensions were prepared from PBMCs, spleen (n = 4 at each time point) and tumor tissues (n = 2 at each time point) of these mice on 5 days, 10 days and 20 days after the transfer and subjected to flow cytometry after staining with V450-anti-human CD45 (Clone: HI30), FITC-anti-human TCR Vδ2(Clone: B6), APC-anti-human CD152 (CTLA-4) (Clone: L3D10), PerCP/Cy5.5-anti-human CD366 (Tim-3) (Clone:F38-2E), PerCP/Cy5.5-anti-human CD223 (LAG-3) (Clone: 11C3C65) and analyzed after gated on $hCD45^{+}V\delta2^{+}$ cells.  APC-mouse IgG1, κ (Clone: MOPC-21), PE-mouse IgG1, κ (Clone: MOPC-21), PerCP/Cy5.5-mouse IgG1, κ (Clone: MOPC-21) were used as isotype controls to obtain delta changes of MFI.

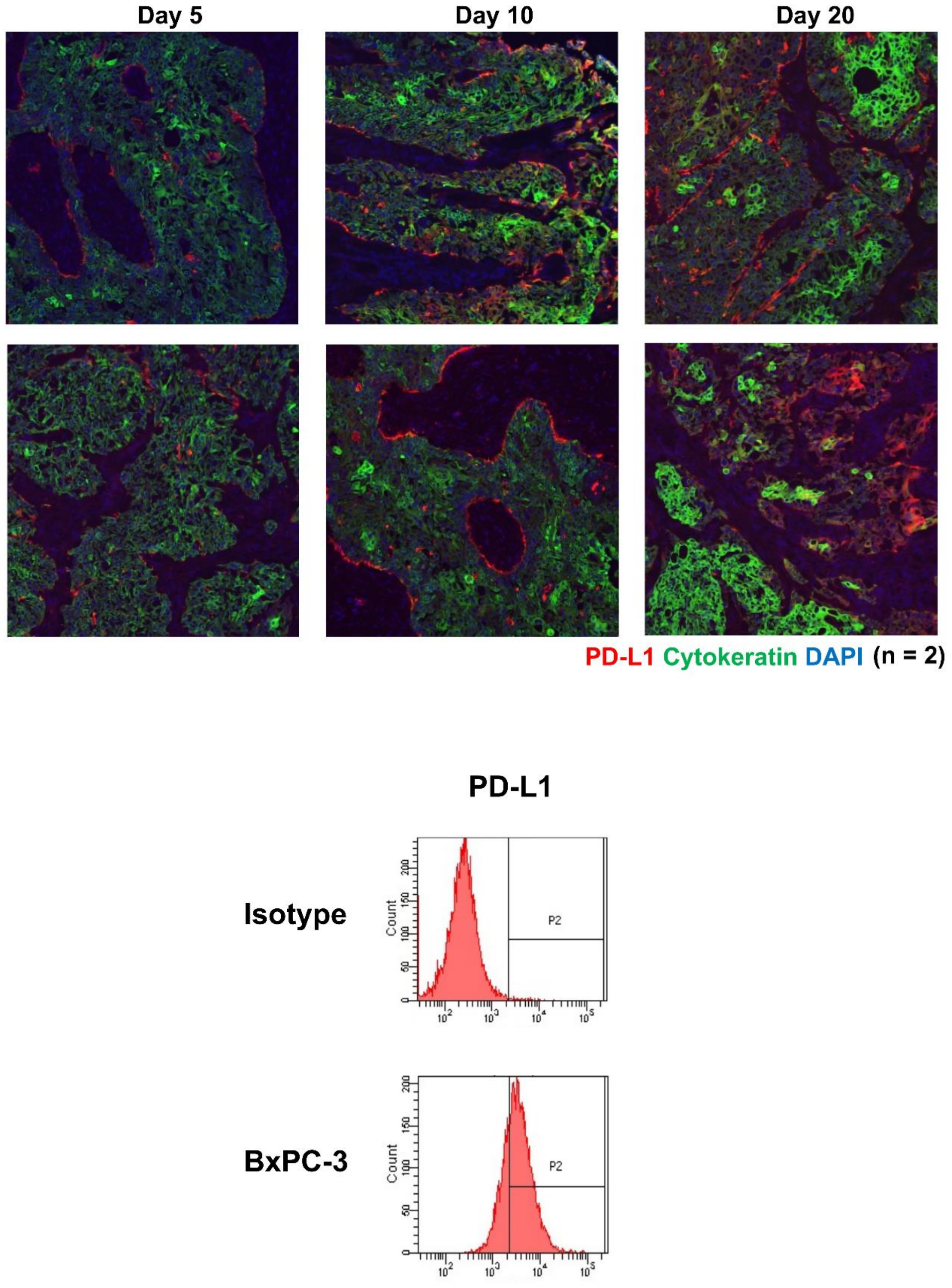


**Figure S6. Expression immunoinhibitory molecules**

(A) Tumor tissues collected from mice in Figure S4B were subjected to fluorescence IHC for PD-L1 expression using PE-anti-human PD-L1(Clone: 29E.2A.3)(red), pan-Cytokeratin (Clone: AE1/AE3) (green) and DAPI (blue). (B) Expression of PD-L1 on BxPC-3 cultured in vitro.

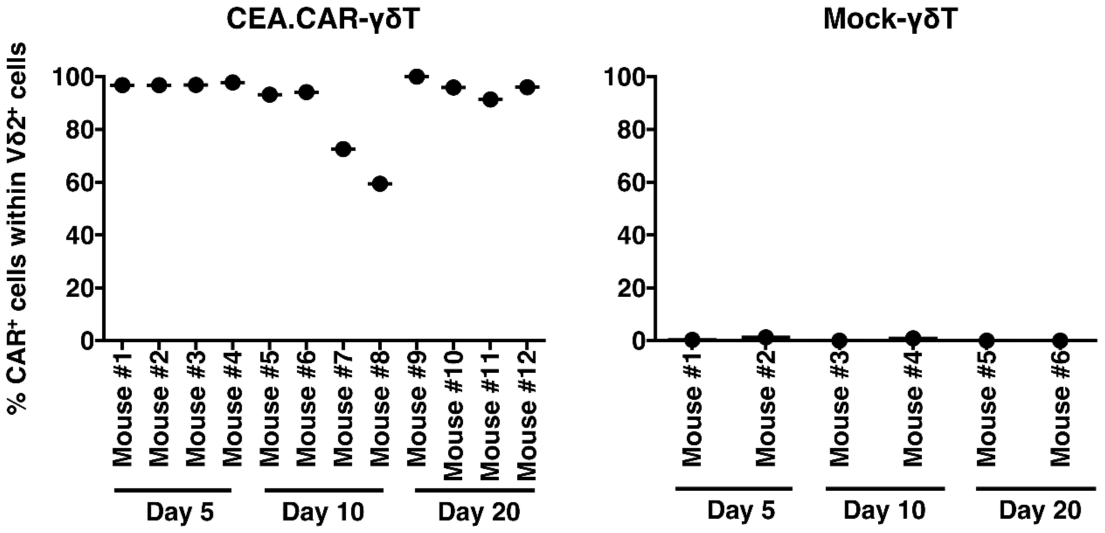


**Figure S7. Maintenance of CAR expression on transferred CEA.CAR-γδT cells.**

NOG mice were inoculated s.c. with BxPC-3 ($4 \times 10^6$ cells) on day 0 followed by i.v. injection with CEA.CAR-γδ T cells ($5 \times 10^6$ cells) (n = 4) or Mock-γδ T cells ($5 \times 10^6$ cells) (n = 2) on day 7. PBMCs were collected from these mice (CEA.CAR-γδ T cells at indicate time points and subjected to flow cytometry after staining with biotinylated-

CEA followed by streptavidin PE-conjugated and FITC-anti-human TCR Vδ2 (Clone: B6). Percentages of $CAR^+$ cells within $V\delta2^+$ cells are shown.

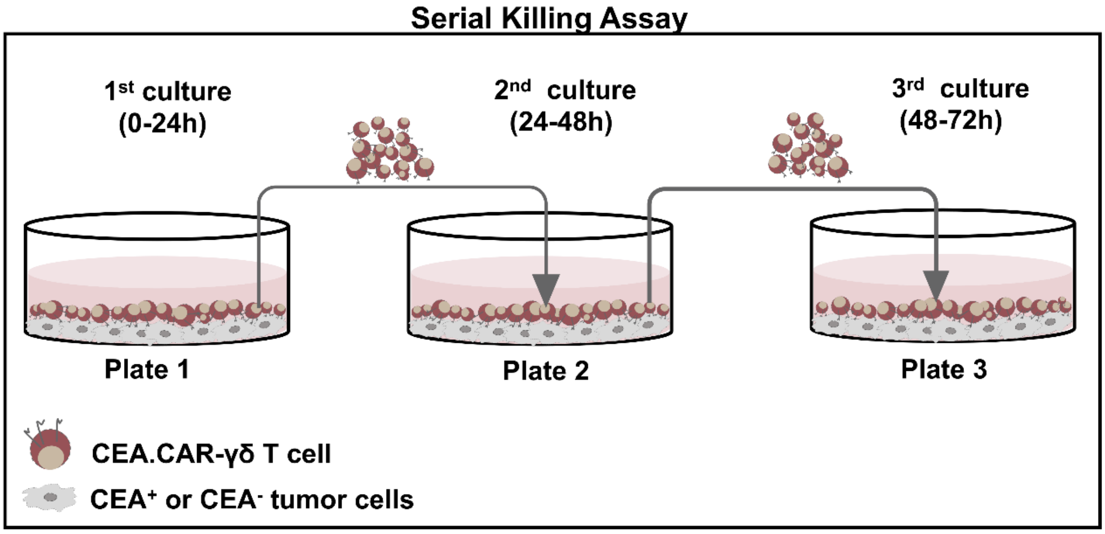


**Figure S8. Schematic diagram of CEA.CAR-γδT cells serial killing assay.**

A schematic representation of CEA.CAR-γδ T cells serial killing protocol. $CEA^+$ (BxPC-3) or $CEA^-$ (MIA Paca-2) tumor cells were seeded at 1st, 2nd, and 3rd round co-culture plate. CEA.CAR-γδ T cells were added and co-cultured for 24 hours (initial co-culture). After 24 hours, CEA.CAR-γδ T cells from the 1st culture plate were harvested and transferred to a new well with previously seeded tumor cells at 24-hour interval for subsequent rounds of killing (2nd and 3rd).
